# Construction of recombinant *sestc Saccharomyces cerevisiae* for consolidated bioprocessing, cellulase characterization, and ethanol production by in situ fermentation

**DOI:** 10.1007/s13205-016-0512-9

**Published:** 2016-09-03

**Authors:** Peizhou Yang, Haifeng Zhang, Shaotong Jiang

**Affiliations:** The Key Laboratory for Agricultural Products Processing of Anhui Province, College of Food Science and Technology, Hefei University of Technology, Tunxi Road 193, Hefei, 230009 Anhui China

**Keywords:** *Saccharomyces cerevisiae*, Cellulase, *Sestc* gene, Engineering strain, Ethanol, In situ fermentation

## Abstract

Bioethanol is an important oil substitute produced by the sugar fermentation process. To improve the efficiency of cellulase expression of *Saccharomyces cerevisiae*, a eukaryotic expression vector harboring a single-enzyme-system-three-cellulase gene (*sestc*) was integrated into the *S. cerevisiae* genome by the protoplast method. Using PCR screening, RT-PCR, and “transparent circle” detection, several recombinant *S. cerevisiae* strains, capable of efficiently expressing the heterogeneous cellulase, were selected. The total activity of cellulase, endo-β-D-glucanase, exo-β-D-glucanase, and xylanase of the recombinant *S. cerevisiae* transformant (designated number 14) was 1.1, 378, 1.44, and 164 U ml^−1^, respectively, which was 27.5-, 63-, 24-, and 19-fold higher than that of the wild-type strain. The concentration of ethanol produced by the engineered *S. cerevisiae* strain was 8.1 gl^−1^, with wheat bran as the carbon source, under submerged conditions; this was 57.86-fold higher than that produced by the wild-type strain (0.14 gl^−1^).

## Introduction

In recent years, with the dramatic increase in demand for petroleum, natural gas, coal, and other non-renewable energy sources has necessitated the development of solutions to the fossil energy crisis (Saini et al. 2015). Biomass-derived fuel obtained by converting lignocellulose into ethanol represents an attractive alternative source of energy. This process requires the application of three key technologies: pretreatment of raw material, saccharification, and fermentation. The saccharification process is highly dependent on the synergistic actions of cellulase and hemicellulase. One of the main components of enzymatic hydrolysis production is glucose, which may be used for ethanol production by the yeast *Saccharomyces cerevisiae* (Tiboni et al. 2014).

Wild-type *S. cerevisiae* possesses extremely low cellulase activity under general conditions. The bottleneck of simultaneous glycosylation and fermentation may be overcome by increasing the cellulase activity of *S. cerevisiae* (Zerva et al. 2014). Cellulase activity can be improved by two techniques: the first method involves the integration of a single cellulase system gene, e.g. *cbh* (Haan et al. 2013), e.g. (Baek et al. 2012), and *bgl* (Tang et al. 2013) were individually integrated into the *S. cerevisiae* chromosome. In practice, the degradation of lignocellulose complexes via the overexpression of a single cellulase system gene has proven difficult. The second method, which involves co-expression of multiple cellulase genes in *S. cerevisiae*, has been found to be highly effective in degrading the hard structure of lignocelluloses. Several combinations of cellulase-encoding genes such as *Trichoderma reesei egi*/*Saccharomycopsis fibuligera bgli* (Haan et al. 2007), *T. reesei* e.g. II/*Aspergillus aculeatus bgl* (Fujita et al. 2002), three β-glucosidase (BGL) genes and two endoglucanase (EG) genes from *Aspergillus oryzae* (Kotaka et al. 2008), *Endomycopsis fibuliger bgl1*/*Butyrivibrio fibrisolvens end1*/*Phanerochaete chrysosporium cbh1*/*Ruminococcus flavefaciens cell* (Rensburg et al. 1998), and *T. reesei egII*/*T. reesei cbhII*/*A. aculeatus bgl1* (Fujita et al. 2004) was used to increase the efficiency of decomposition of recalcitrant lignocelluloses. Because it is difficult to maintain the expression of several cellulase genes at an identical level, the use of a cellulase cocktail should enable optimal decomposition (Yamada et al. 1978). Therefore, the development of an engineered strain harboring a single enzyme system that incorporates the activity of multiple cellulases would be extremely useful to achieve synergistic and efficient degradation of cellulose.

The conversion of lignocellulosic biomass into fuels by in situ fermentation is promising. The degradation of lignocellulosic material, composed of cellulose and hemicelluloses, requires the action of multiple enzymes capable of hydrolyzing the recalcitrant lignocellulose structure into reducing sugars. These cellulolytic and xylanolytic enzymes include EGs, exoglucanases, BGLs, and xylanase, which cleave cellobiose or oligosaccharide units into glucose and xylose monomers. The consolidation of several processes involving cellulase production, lignocellulose hydrolysis, and fermentation during bioprocessing is difficult, which leads to high costs of biomass production and low economic efficiency. Consolidated bioprocessing, in which these three key processes are combined into a single step, represents a promising alternative approach. The strategies involved in consolidated bioprocessing include the native cellulolytic strategy and the recombinant cellulolytic strategy (Lynd et al. 2005). The feasibility of consolidated bioprocessing using a recombinant strategy depends on the combination of fermentation and reduction in the reactivity of saccharification of the substrate (Walsum and Lynd 1998). To overcome this bottleneck, cellulases, xylanases, and amylases have been expressed in various *S. cerevisiae* strains (Katahira et al. 2004). In addition, engineered *S. cerevisiae* strains have been used for the functional expression of several cellobiohydrolases (Hong et al. 2003).

The single-enzyme-system-three-cellulase gene (*sestc*), isolated from *Ampullaria gigas* Spix, encodes a three-cellulase system comprising the endo-beta-1,4-glucanase, exo-beta-1,4-glucanase, and xylanase enzymes (Shujie et al. 2009), and was also found to be a multifunctional cellulase gene. In this study, an expression vector harboring the *sestc* gene was integrated into the *S. cerevisiae* chromosome using a PEG-mediated protoplast genetic integration technique. The characteristics of the extracellular enzyme produced by successful transformants were investigated and the activity of the three-cellulase system (comprising endo-beta-1,4-glucanase, exo-beta-1,4-glucanase, and xylanase) was studied. In addition, the production of ethanol was investigated under submerged conditions with wheat bran as carbon source.

## Materials and methods

### Genes, expression vector, strain, and lytic enzyme

**Fig. 1:**
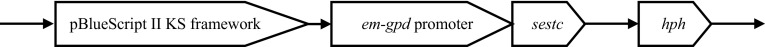
The structure of the reading frame of the expression vector

The reading frame of the eukaryotic expression plasmid was based on that of the pBlueScript II KS vector (Fig. 1). The glyceraldehyde-3-phosphate dehydrogenase gene (*gpd*) promoter, isolated from Enoki Mushroom (*em*-*gpd*), was adopted (Yang et al. 2011). The *sestc* gene was isolated from the stomach tissue of *A. gigas*. The hygromycin resistance gene (*hph*) was used as the selectable marker gene. The industrial strain of *S. cerevisiae* was maintained at a laboratory at the College of Food Science and Engineering, Hefei University of Technology. The lytic enzyme for protoplast preparation was obtained from the Microorganism Preservation Center of Guangdong province.

### Genetic integration and molecular identification

The expression vector was integrated into the *S. cerevisiae* chromosome randomly using the protoplast transformation method. A standard colony of *S. cerevisiae* was inoculated into 50 ml of YPD broth medium obtained from Sangon Biotech containing 1 % yeast powder, 2 % peptone, and 2 % glucose, at 30 °C, with shaking at 180 rpm, for 48 h in a 250 ml Erlenmeyer flask. Then, 5 ml of fermentation broth was removed and centrifuged at 800 rpm at 4 °C for 5 min using a centrifuge manufactured by Backman Company; the solid cells were collected and washed three times with ultrapure water. Next, 1 ml of 2 % (wv^−1^) lywallzyme was added to a sterilized 10 ml Eppendorf tube manufactured by Haimen United Laboratory Equipment Development Co., Ltd. The broth was incubated at 30 °C, with shaking at 80 rpm for 1 h, and the *S. cerevisiae* protoplast thus obtained was observed using an optical microscope (Olympus Microscope CX21) with a magnification of ×400. When the protoplast concentration reached 80 %, the enzymatic reaction was gradually decreased through dilution in threefold of the solution volume using sorbitol/CaCl_2_ (S/C) solution prepared with 0.6 mol l^−1^ KCl pre-cooling solution, 1 M sorbitol, and 50 mmol l^−1^ CaCl_2_. Three layers of qualitative filter paper manufactured by Yancheng Yongkang Lab Instruments Factory, China were used to remove the cell debris and other large fragments produced by enzymatic hydrolysis. The filtrate was centrifuged at 800 g rpm in 4 °C for 10 min using a Beckman Coulter Allegra 64R refrigerated centrifuge to precipitate the protoplast, and the supernatant was discarded. The collected cells were washed and precipitated using S/C solution. After centrifugation, the protoplasts were suspended in 50 μl of S/C solution. The expression plasmid (10 μg) was added to a protoplast suspension of 100 μl; this was supplemented with 50 μl of polyethylene glycol (PEG) buffer and gently mixed using a pipette. After placing in an ice bath for 20 min, 1 ml of PEG buffer (prepared with 25 % PEG8000, 50 mmol l^−1^ CaCl_2_, and 10 mmol l^−1^ Tris HCl, at pH 7.5) was added to the reaction mix. The reaction was incubated at 20 °C for 5 min; then, 2 ml of S/C solution was added and the reaction system was gently mixed. After a 10× dilution, the solution was evenly coated on to solid regeneration medium containing 0.3 % yeast powder, 1 % peptone, 2 % glucose, 0.6 mol l^−1^ MgSO_4_, and 1.8 % agar, and incubated for 24–72 h at 30 °C. Then, a single colony was picked for analysis by PCR. The primers used for amplification of partial fragments of the *sestc* gene, by PCR and RT-PCR, were as follows: primer R: 5′-GCTTCAGTCAAGCGCATGCC-3′; primer F: 5′-GTCGGCGGCGTGTGCGATACG-3′. The PCR reactions were performed in a 30 μl total volume containing 20 μl of sterile deionised water, 3 μl of 10 × Taq DNA polymerase, 3 μl of 2 mM dNTP, 1.5 μl of 50 mM MgCl_2_; 2 μl of 10 pmol μl^−1^ F + R primer stock; 0.4 μl of DNA (approximately 20 ng per reaction minimum), 0.25 μl of Taq DNA polymerase. The PCR program was as follows: step 1 at 94 °C for 10 min, step 2 at 94 °C for 40 s, step 3 at 64 °C for 30 s, step 4 at 72 °C for 1 min, step 5 repeat step 2 × 3 times, step 6 at 94 °C for 40 s, step 7 at 56 °C for 30 s, step 8 at 72 °C for 1 min, step 9 at 6 × 35 times, step 10 at 72 °C for 10 min, and step 11 at 4 °C indefinitely.

### Calculation of sizes of transparent circles as a measure of the strength of cellulase activity

**Fig. 2:**
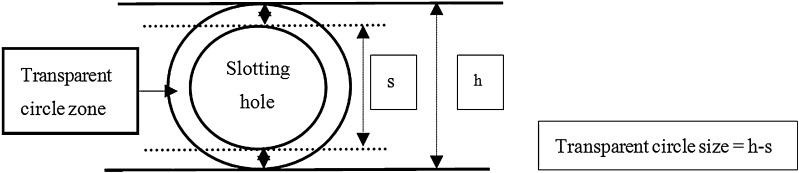
The calculation of the sizes of the *transparent circle* formed in the solid culture medium

Solid sodium carboxymethyl cellulose medium consisting of 0.2 % NaNO_3_, 0.1 % K_2_HPO_4_, 0.05 % MgSO_4_, 0.05 % KCl, 0.2 % sodium carboxymethyl cellulose, 0.02 % peptone, and 1.7 % agar powder, was prepared. The surface of the solid sodium carboxymethyl cellulose medium containing Petri dish was evenly divided into four zones using a marker pen. A single hole of 5 mm diameter was made in each zone. Then, 5 ml of culture medium was centrifuged at 3577.6×*g* at 4 °C for 15 min in 10 ml centrifuge tubes. The supernatant (100 μl) was absorbed and added to the previously made hole in the solid culture medium. This volume was added to each hole. After incubation for 5 h at 30 °C, iodine solution, containing 6 % potassium iodide and 3 % iodine, was prepared. The formation of transparent circles, which indicated cellulase activity, was observed for a period of 15 min. The formation of transparent circles indicated the production of reducing sugars owing to the decomposition of sodium carboxymethyl cellulose by cellulase; the larger the transparent circle formed, the stronger the cellulase activity. The sizes of the transparent circles were calculated by determining the difference between the diameter of the outer transparent circle (h) and that of the inner slotting holes (s) (Fig. 2). This method enabled the identification of colonies of true transformants harboring the cellulase gene.

### Fermentation and cellulase expression

The colonies of wild-type *S. cerevisiae* and transformants were separately inoculated into 50 ml of seed media (prepared with 10 gl^−1^ sodium carboxymethyl cellulose, 20 gl^−1^ peptone, and 10 gl^−1^ yeast powder sterilized at 121 °C for 20 min) in a 250 ml Erlenmeyer flask at 30 °C with shaking at 150 rpm for 36 h. Then, 3 ml of the media was taken out and transferred into 100 ml of liquid fermentation medium, prepared with 20 gl^−1^ wheat bran, 20 gl^−1^ peptone, 10 gl^−1^ yeast powder, and 5 gl^−1^ glucose, sterilized at 121 °C for 20 min, in a 250 ml Erlenmeyer flask. The culture was incubated for 24 h at 30 °C with shaking at 150 rpm. Next, 3 ml of the culture was collected every 6 h to evaluate the total activity of cellulase, endo-beta—D-glucosidase, exo-beta- glucosidase, and xylanase.

### Characterization of the cellulase enzyme produced by the transformants

The transformants forming the largest transparent circles, as described in Sect. “Calculation of sizes of transparent circles as a measure of the strength of cellulase activity”, were selected for characterization of the total activity of cellulase, endo-β-D-glucosidase, exo-β-D-glucosidase, and xylanase. Cellulase characteristics, such as the optimum reaction temperature and pH, thermal and acid stability, and the effects of metal ions such as K^+^, Mn^2+^, Zn^2+^, Cu^2+^, Fe^2+^, and Fe^3+^, were investigated.

### Measurement of cellulase and xylanase activity

The activities of total cellulase, endo-β-D-glucosidase, exo-β-D-glucosidase, and xylanase were measured using filter paper, sodium carboxymethyl cellulose, microcrystalline cellulose, and xylan as substrates, respectively. The dinitrosalicylic acid (DNS) method was used, and one enzymatic activity unit was defined as that required for the generation of 1 μmol of glucose or xylose per min in 1 ml of enzyme solution (IUPAC 1987; Zhang et al. 2009; Nummi et al. 1985).

### Preparation of fermentation media and in situ fermentation for ethanol production

Using wild-type *S. cerevisiae* as the control, a transformant designated number 14 was used to detect ethanol production by submerged fermentation. The wild-type colonies and those of transformant number 14 were inoculated into a 250 ml Erlenmeyer flask containing 50 ml seed media [which consisted of 5 % glucose, 2 % peptone, 0.5 % (NH_4_)_2_SO_4_, and 0.1 % MgSO_4_] and incubated at 30 °C for 36 h at 150 rpm. Then, 10 ml of broth was added to a 250 ml Erlenmeyer flask containing 50 ml of fermentation media [adjusted to pH 6.0, containing 4 g of rice straw, 1 g of wheat bran, 0.4 g of (NH_4_)_2_SO_4_, 0.6 g of KH_2_PO_4_, 0.1 g of CaCl_2_, 0.1 g of MgSO_4_, 0.1 g of MnSO_4_, 0.1 g of ZnSO_4_, and 0.1 g of Cocl_2_]. After 48 h of incubation at 30 °C with shaking at 150 rpm, the system temperature of the fermentation culture was increased to 50 °C for 2 h. The temperature was then decreased to 30 °C, while fermentation continued during incubation at 150 rpm for 36 h. The amount of yeast cells in broth were counted to calculate the survival rate of thermal treatment (Rikhvanov et al. 2003). Ethanol concentration was determined using the gas chromatography method (Sree and Sridhar 1999).

## Results and discussion

### Preparation of *S. cerevisiae* protoplasts

**Fig. 3:**
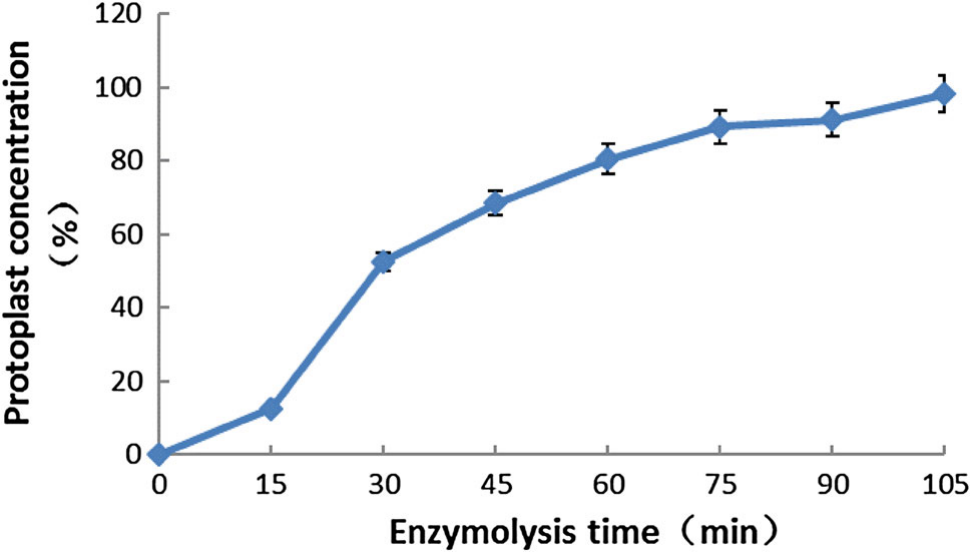
Relationship between protoplast concentration and duration of enzymolysis

The concentration of the *S. cerevisiae* protoplasts were calculated by microscopy. The *S. cerevisiae* protoplast concentration was nearly 80 % after a 60 min treatment of lytic enzyme. After treatment for over 105 min, the protoplast concentration exceeded 90 % (Fig. 3).

**Fig. 4:**
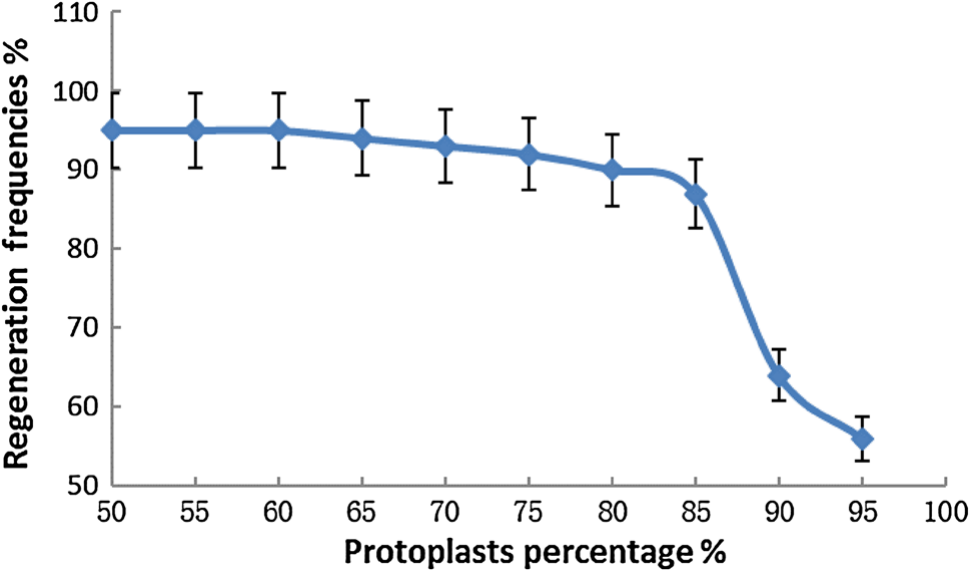
Relationship between regeneration frequencies and protoplasts concentration

Figure 4 shows the relationship between regeneration frequency and protoplast concentration for *S. cerevisiae*. When the protoplast concentration reached 90 %, the regeneration frequency decreased to 64 %. At higher concentrations, the protoplasts were more easily broken under a given centrifugation pressure. In addition, the protoplasts were easily damaged or torn by pipettes during transformation and mixing. Insufficient digestion by the lytic enzyme and excessive treatment both led to a decrease in efficiency of genetic transformation. In this study, to avoid severe injury to protoplasts due to excessive enzymolysis, enzymatic hydrolysis was terminated after 60 min. Residual lytic enzyme attached to yeast cells was removed by centrifugation and washing. The regeneration frequency is a key index for genetic transformation of microorganisms, and optimal conditions for protoplast formation vary between species and strains (Zhang et al. 2016). For *S. cerevisiae*, reversion frequencies reached 50 % on agar-solidified media (Svoboda 1966). Sorbitol (1 M) was applied to regeneration agar, and a PEG-mediated transformation method was used to improve the transformation efficiency (Zhang et al. 2016). In addition to supplementation with sorbitol and PEG, CaCl_2_ and Tris HCl were used to improve the efficiency of protoplast regeneration.

### Screening of hygromycin concentration and *S. cerevisiae* protoplast regeneration

The growth of *S. cerevisiae* on YPD-hygromycin B media was investigated. *S. cerevisiae* is generally sensitive to hygromycin B. The numbers of colonies on solid media, with the addition of various hygromycin B concentrations, were counted (Table 1). Protoplast growth of *S. cerevisiae* was seriously inhibited when the concentration of hygromycin B exceeded 200 mg l^−1^. In view of the inhibitory effect of hygromycin B on *S. cerevisiae* protoplast activity, a hygromycin B concentration of 200 mg l^−1^ was used to screen transformants harboring the *sestc* expression vector. Bi-functional expression vectors were constructed in which the phosphoglycerate kinase gene was fused to the hygromycin B resistance gene (Kaster et al. 1984). A hygromycin B concentration of 200 mg l^−1^ was sufficient to inhibit the growth of *S. cerevisiae* in solid agar plates. In this study, *S. cerevisiae* cells harboring the expression vector harboring the *sestc* gene and the hygromycin B resistance gene were found to be resistant to high levels of hygromycin B.

**Table 1:** Effect of hygromycin concentration on the regeneration of *S. cerevisiae* protoplasts

Hygromycin B concentration (mg l^−1^)	Colony (numbers)
0	362 ± 47
50	211 ± 34
100	163 ± 16
150	44 ± 7
200	0
250	0

### Screening of transformants and molecular identification

**Fig. 5:**
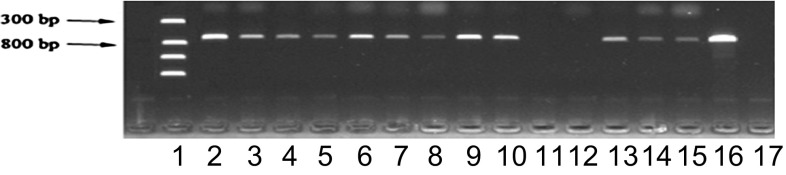
Electrophoretic resolution of PCR-amplified DNA extracted from the transformant colonies *Lane 1* DNA marker, *Lanes 2*–*15* transformants, *Lane 16* positive control, *Lane 17* negative control

PCR amplification was used for analysis of DNA extracted from the transformants (Fig. 5). The electrophoresis data show that the entire genome was amplified. In lanes 11 and 12, no DNA was visualized, whereas other lanes showed bright bands. Colonies corresponding to PCR products in lanes 2, 6, 9, and 10, in which bright bands could be visualized, were selected for further analysis. The PCR results indicated that the percentage of positive transformants was 85.4 %, which represented the vast majority of colonies on solid screening media.

**Fig. 6:**
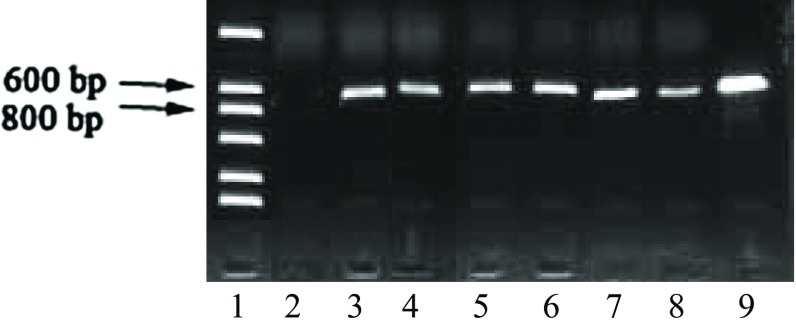
Detection of *sestc* expression using RT-PCR. *Lane 1* marker, *3*–*8* transformants, *Lane 9* positive control, *Lane 2* negative control

Reverse transcriptase-polymerase chain reaction (RT-PCR) was used to verify the transformants, based on the analysis of *sestc* expression (Fig. 6). After the preparation of RNA and synthesis of cDNA, PCR was performed to amplify the cDNA product using the designed primers. Positive transformants were identified by agarose gel electrophoresis, which indicated that 90.1 % of the transformants were positive. RT-PCR has been previously used to assess the expression of candidate genes in *S. cerevisiae* (Basso et al. 2015). In addition, RT-PCR has been used for the evaluation of a vector for multi-copy integration into the *S. cerevisiae* chromosome and to confirm high-copy integration events (Tripathi et al. 2012). In this study, RT-PCR was used for further verification of the positive transformant.

### The “transparent circle” method

**Fig. 7:**
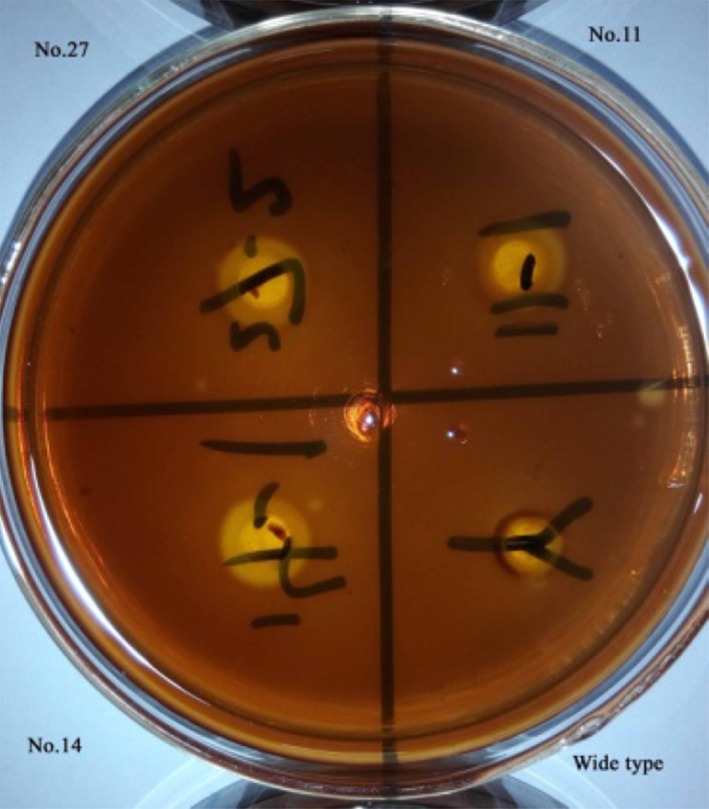
Transparent circle on a stained sodium carboxymethyl cellulose plate on which *S. cerevisiae* transformants and the wild-type strain had been cultured

To evaluate the effect of transformation and expression of the cellulase-encoding gene further, the transformants were grown on solid sodium carboxymethyl cellulose medium. After incubation for 6–8 h, results indicated the formation of transparent circles of various sizes on the Petri dish. In Fig. 7, the bright middle zone was perforated using a puncher to form a hole. The transparent circle formed due to the activity of cellulase was viewed as an innermost bright zone surrounding the hole. The darker outermost zone around the hole represented the region of the solid medium in which no enzymatic activity had occurred. Figure 7 shows the sizes of the transparent circle sizes for recombinant transformants numbers 11, 14, and 27 as well as the wild-type strain. The transformants were found to be capable of expressing cellulase, whereas the wild-type strain was not, as indicated by the absence of a transparent circle in solid medium in which the latter was grown. The “transparent circle” method has been used to isolate strains capable of degrading straw with high efficiency (Philippidis and Hatzis 1997). In this study, this method was used to investigate the ability of the present strains to produce cellulase.

The sizes of the transparent circle were calculated based on a formula (Fig. 2). The transparent circle produced by the wild-type strain was small, whereas those produced by the transformants were significantly larger (Table 2); the largest of these, which measured 7.65 mm in diameter and was 63.8-fold larger than that of the wild-type, was produced by transformant number 14. The transparent circle experiment showed that the cellulase activity of the transformants was significantly higher than that of the wild-type strain, confirming that the *sestc* gene is highly expressed in *S. cerevisiae* and that the cellulase enzyme encoded by this gene is secreted from the cell.

**Table 2:** Sizes of the transparent circles formed in the *S. cerevisiae* transformant culture and wild-type culture on solid medium

Strain	Size of transparent circle (mm)	Fold–difference compared with that of wild-type
Transformant number 11	4.21 ± 0.54	35.1
Transformant number 14	7.65 ± 0.98	63.8
Transformant number 27	4.55 ± 0.48	37.9
Transformant number 36	4.62 ± 0.35	38.5
Transformant number 37	5.02 ± 0.41	41.8
Transformant number 59	5.59 ± 0.55	46.6
Transformant number 60	6.23 ± 0.28	51.9
Transformant number 66	5.68 ± 0.45	47.3
Transformant number 69	6.84 ± 0.87	57
Wild-type	0.12 ± 0.01	–

### Comparison of the expression of the cellulase system by the transformant grown in fermentation culture and by the wild-type strain

**Fig. 8:**
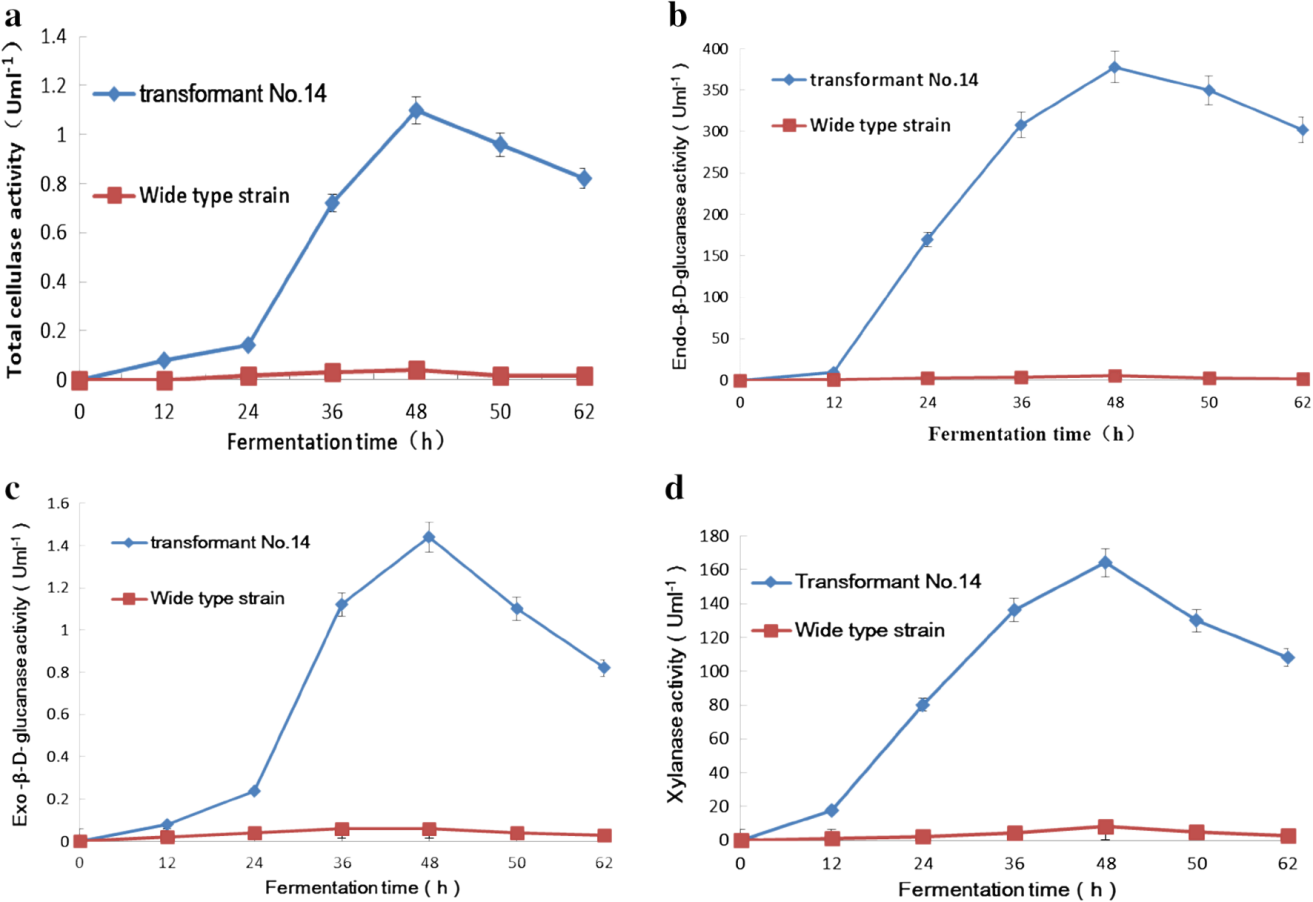
Lignocellulase activity of recombinant number 14 and the wild-type strain. **a** Total cellulase activity; **b** Endo-β-D-glucanase activity; **c** Exo-β-D-glucanase activity; **d** xylanase activity

*Saccharomyces cerevisiae* strains capable of simultaneously producing and secreting several heterologous cellulases are highly useful for applications such as consolidated bioprocessing. In a previous study, two native *S. cerevisiae* genes, *PSE1* and *SOD1*, were overexpressed under the transcriptional control of the constitutive *pgk1* promoter. A dramatic increase (of 447 %) in β-glucosidase secretion was observed in the engineered *S. cerevisiae* strain (Kroukamp et al. 2013). However, the method used resulted in enzyme-specific effects, induced by Cel3A secretion, whose activity was greater than that of the other cellulases. To maximize heterologous protein secretion for consolidated bioprocessing, the integration of genes encoding cellulases amenable to expression in engineered *S. cerevisiae* is imperative (Kroukamp et al. 2013). The over-expression of a single cellulase system in the native *S. cerevisiae* strain did not result in enhancement of the total cellulase activity, as secretion was a limiting factor for consolidated bioprocessing in this engineered yeast (Mood et al. 2013). The reporter proteins *S. fibuligera* BGL was used to investigate the secretion of recombinant proteins in *S. cerevisiae* (Al-Baghdadi 2003). Moderate to low secretion levels were observed for CBHs (Zaldivar et al. 2001), BGLs (Elia et al. 2008) and EGs (Cavka et al. 2014). Therefore, the production of cellulolytic enzymes via the expression of recombinant cellulase genes was particularly challenging. In this study, assessment of the activity of cellulase enzymes indicated that the heterologous cellulase *sestc* gene was expressed with high efficiency under the control of the *em*-*gpd* promoter. In this study, transformant number 14 was selected to analyze the expression of cellulase and ethanol production. After fermentation for 48 h, the cellulase activity, with wheat bran as fermentation substrate, reached a peak. For *S. cerevisiae* transformant number 14, following 48 h of fermentation, the total activity of cellulase (as determined using the filter paper method), endo-β-D-glucanase, exo-β-D-glucanase, and xylanase activity was 1.1, 378, 1.44, and 164 U ml^−1^, respectively, which was 27.5-fold (compared with 0.2 U ml^−1^), 63-fold (compared with 30 U ml^−1^), 24-fold (compared with 0.3 U ml^−1^), and 19-fold (compared with 43 U ml^−1^) higher than that of the control (Fig. 8).

### Cellulase characteristics of recombinant *S. cerevisiae*

**Fig. 9:**
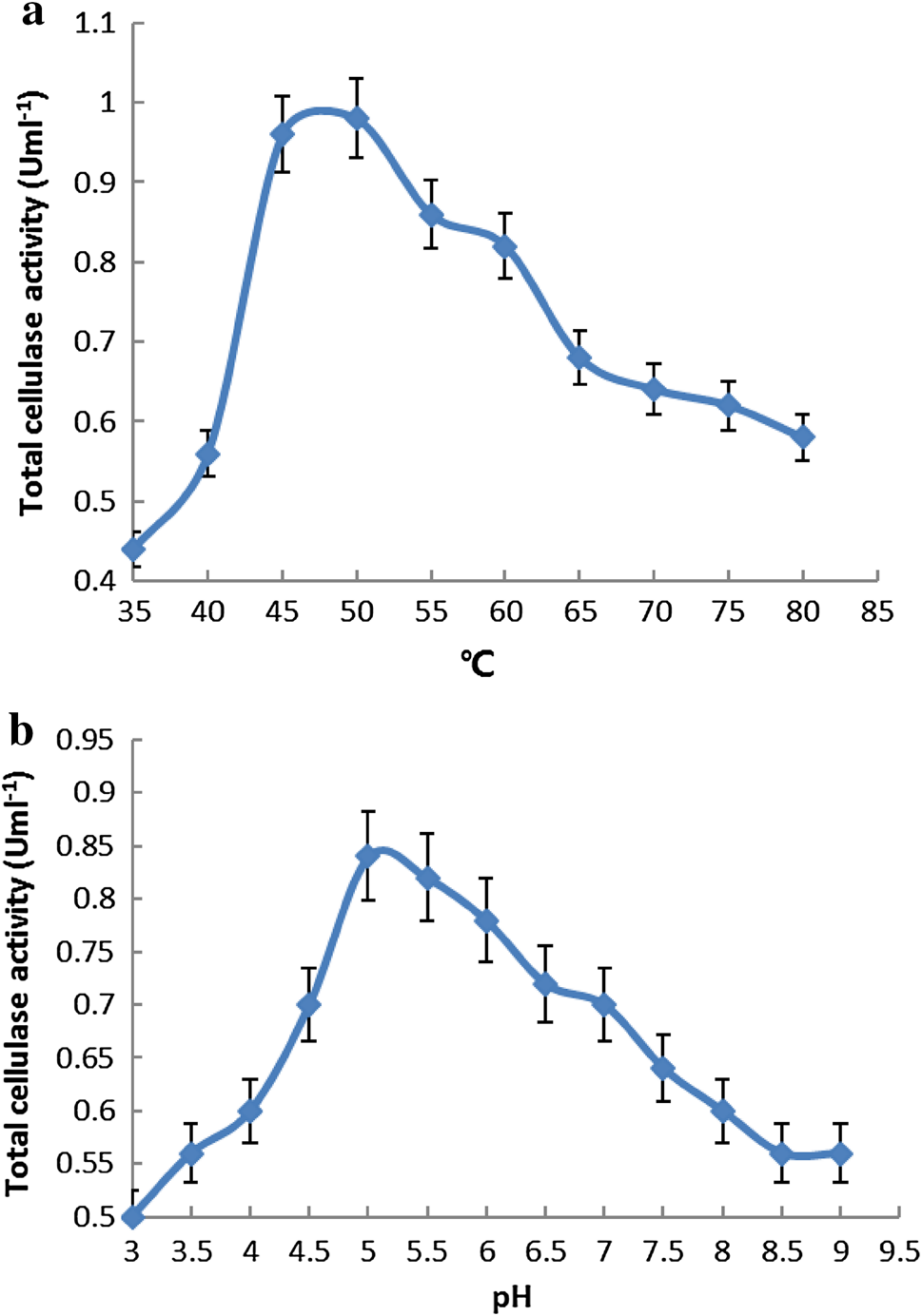
Optimal reaction temperature (**a**) and pH (**b**) for the cellulases of recombinant transformant number 14

The production of bio-ethanol from lignocellulosic biomass represents a promising renewable source of energy. However, the enzymes required for biomass conversion are expensive to obtain. To utilize cellulolytic enzymes comprising a single enzymatic system for such processes, the cellulase and xylanase enzymatic cocktail must be characterized in terms of temperature and pH (Taneda et al. 2012). In this study, the catalytic efficiency of the engineered *S. cerevisiae* strain, which harbored a heterologous single enzyme system comprising three cellulase activities, required the synergistic interaction of the cellulolytic enzymes. The recombinant *S. cerevisiae* transformant number 14, which efficiently expressed the *sestc* gene, was used to investigate the cellulase characteristics of the engineered strain. The optimum reaction temperature and pH were 50 °C and pH 5, respectively (Fig. 9a, b). In addition, the exogenous cellulolytic enzyme (*sestc*) was found to be sensitive to external ambient conditions.

**Fig. 10:**
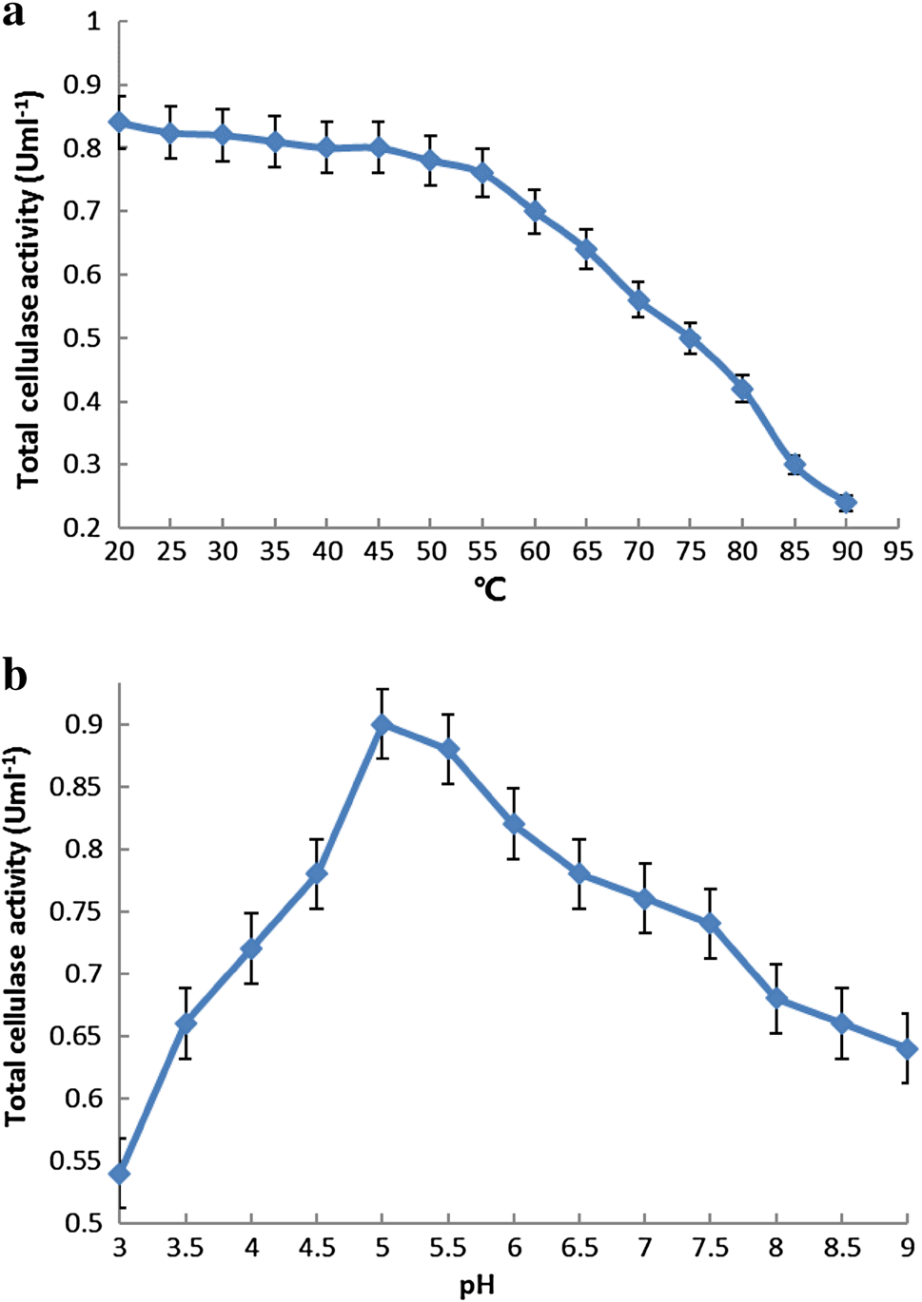
Thermal (**a**) and pH (**b**) stability of recombinant transformant number 14 crude cellulase

It was observed that the final concentration of reducing sugars during hydrolysis decreased with decreasing enzyme activity. The reaction equilibrium was affected by the catalytic efficiency of the enzyme. Thermal instability of enzymes, feedback inhibition, conversion of the substrate into more recalcitrant structure, and deactivation of enzymes affected the thermal and pH stability (Farinas et al. 2010). It was found that the enzyme was stable from 20 to 50 °C; at temperatures above 50 °C, enzyme activity decreased significantly (Fig. 10a), whereas low temperatures (20–50 °C) did not affect the thermal stability of the enzyme. The pH stability was investigated using a citrate buffer solution of pH 3–7, in a water bath, for 5 h at 50 °C. The cellulase activity of the recombinant *S. cerevisiae* transformant number 14 was found to be stable at pH 5 (Fig. 10b).

**Fig. 11:**
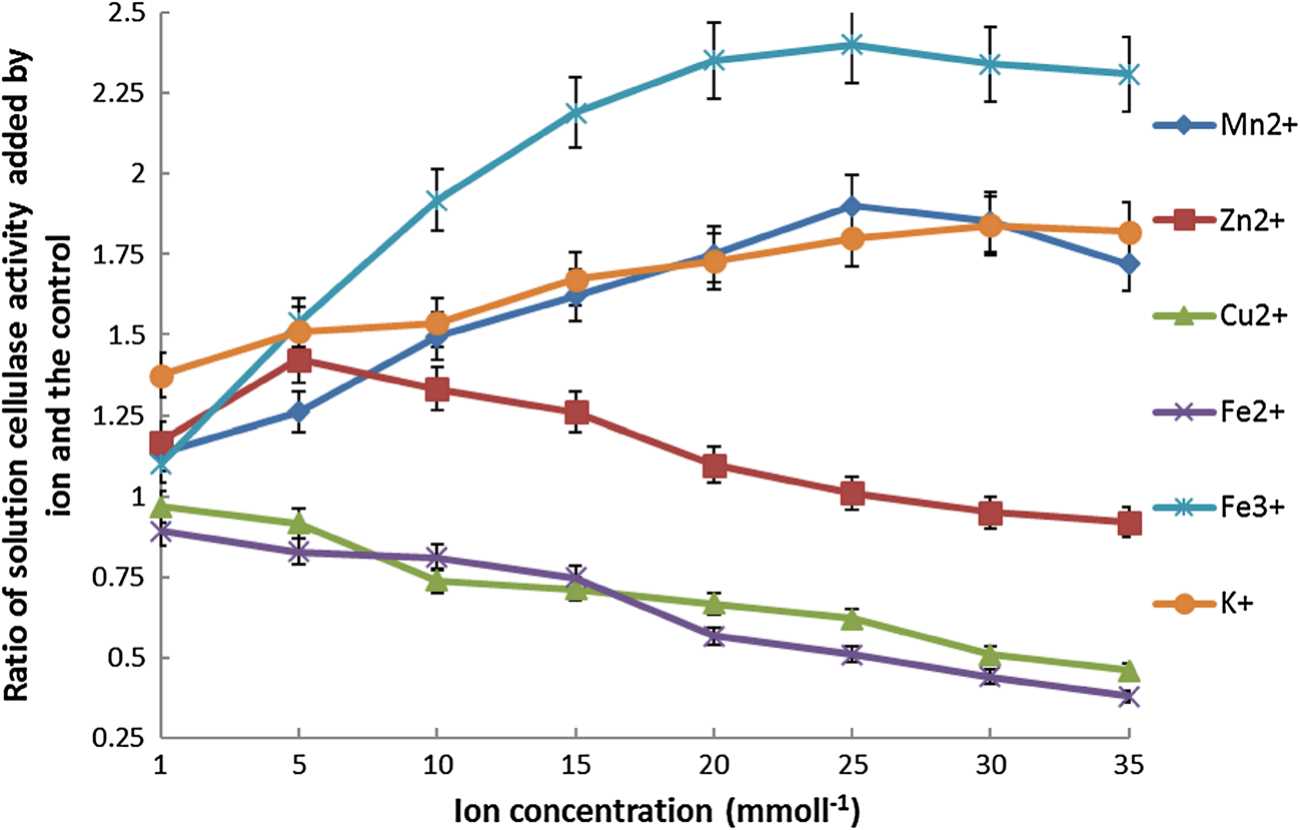
Effects of ion concentration on total cellulase activity of the *S. cerevisiae sestc* transformant

The effects of metal ions, such as K^+^, Mn^2+^, Zn^2+^, Cu^2+^, Fe^2+^, and Fe^3+^, on cellulase activity were investigated (Fig. 11). Cu^2+^ and Fe^2+^ were found to inhibit total cellulase activity. High ionic concentrations of Cu^2+^ and Fe^2+^ led to strong inhibitory effects. Ions K^+^, Mn^2+^, Zn^2+^, and Fe^3+^ were found to increase cellulase activity; the activation effect was the most significant for Fe^3+^. The total cellulase activity was 2.4-fold higher than that of the control for 5 mmol l^−1^ Fe^3+^. The appropriate concentration (1–5 mmol l^−1^) of Zn^2+^ was also observed to increase cellulase activity; at a concentration of 5 mmol l^−1^ of Zn^2+^, the total cellulase activity was 1.42-fold higher than that of the control. At concentrations above 5 mmol l^−1^ Zn^2+^, the cellulase activity increased gradually; however, at excessively high concentrations (30–35 mmol l^−1^) of Zn^2+^, a decline in cellulase activity was observed. At a concentration of 25 mmol l^−1^ of Zn^2+^, the cellulase activity was nearly equal to that of the control. At 0.25 mmol l^−1^, K^+^ and Mn^2+^ produced an equal increase in total cellulase activity. The effect of metal ions on the enzyme was similar to that of acid catalysis, which is involved in oxidation–reduction reactions. In addition, metal ions may exert induction effects on enzyme production due to their electronegativity. The mechanisms underlying inhibition and activation were significantly different. The lower electronegativity of K^+^ is more beneficial for binding of enzyme to substrate, which results in an increase in the activity of the enzyme. Cu^2+^, a heavy metal ion, may cause denaturation of proteins. The toxic effects of high concentrations of heavy metal ions, such as Cu^2+^, are particularly evident.

### In situ fermentation for ethanol production

**Fig. 12:**
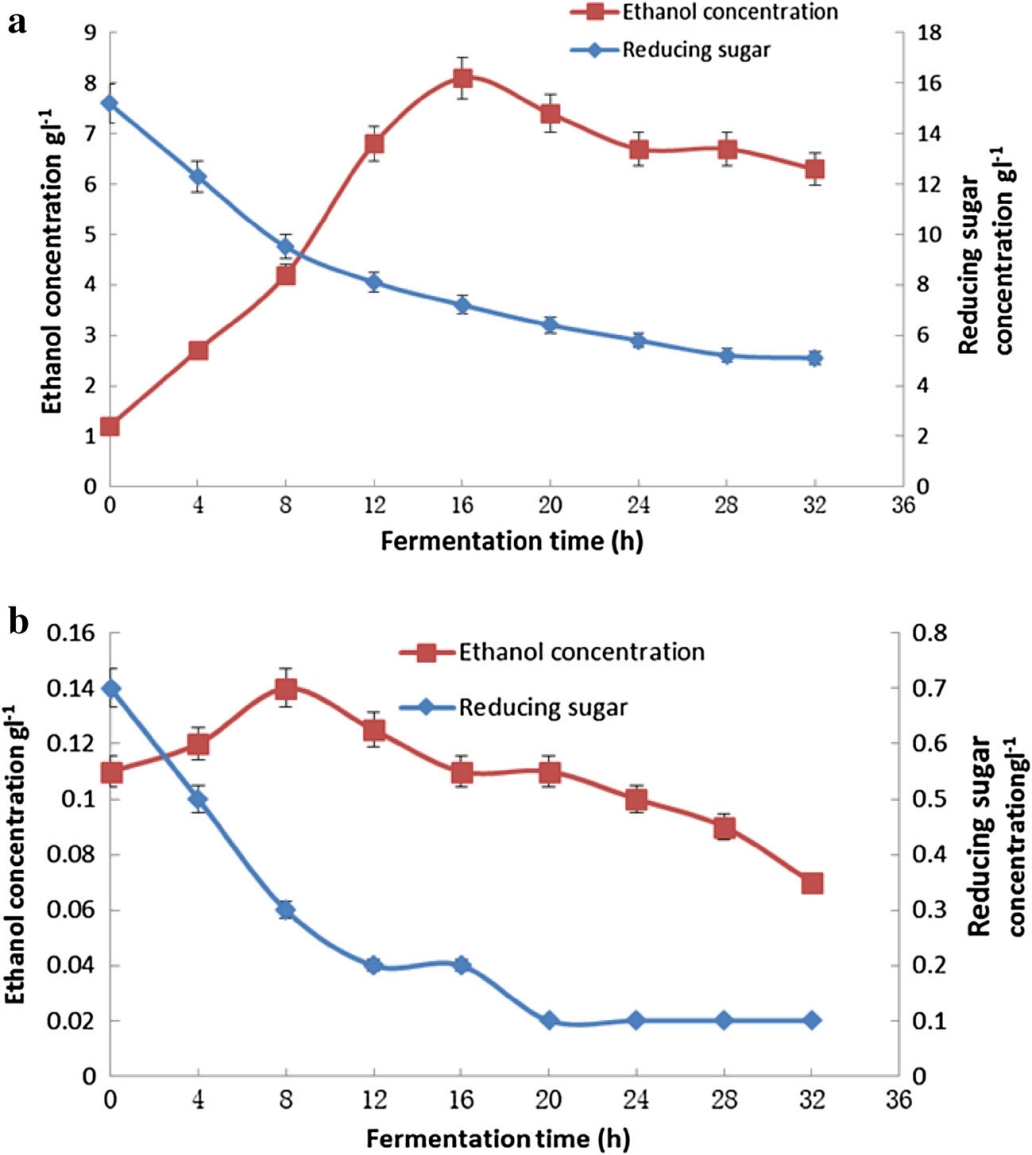
Relationship between the consumption of reducing sugar and ethanol concentration **a** for the transformant; **b** for the wild-type *S. cerevisiae* strain; during subsequent fermentation, ethanol production increased until 8 h of fermentation, then gradually decreased at 8–32 h of fermentation

Reducing sugars were released from the natural substrate when the reaction temperature was 50 °C for 2 h. At 50 °C, microbial activity was inhibited. After thermal treatment, however, the temperature returned to 30 °C; at this temperature, the yeast mainly utilized reducing sugars as the substrate for ethanol production. In this study, ethanol production by the wild-type and recombinant *S. cerevisiae* strains was investigated. For the recombinant *S. cerevisiae* strain, the initial concentration of reducing sugars was higher than that of the wild-type strain, owing to the release of significant amounts of reducing sugars from recalcitrant lignocelluloses crystals due to the action of cellulases. The concentrations of reducing sugar, released by the recombinant *S. cerevisiae* and the wild-type strain, were 12.5 and 0.7 gl^−1^, respectively, and the initial concentrations of ethanol were 1.2 gl^−1^ (Fig. 12a) and 0.11 gl^−1^ (Fig. 12b), respectively. The maximal level of ethanol production was achieved by the recombinant *S. cerevisiae* strain following 16 h of fermentation. Then, the concentration of residual ethanol gradually decreased to 0.3 gl^−1^ following a further 32 h of fermentation. During ethanol production, the concentration of the residual reducing sugar decreased gradually to 5.1 gl^−1^. For the wild-type *S. cerevisiae*, the initial concentration of residual reducing sugar was 0.7 gl^−1^, which was attributed to the low expression of cellulase expressed by this strain. Subsequent fermentation involved the consumption of reducing sugar; the final concentration of which was observed to be about 0.1 gl^−1^ (Fig. 12b).

## Conclusions

An eukaryotic expression vector carrying a single-enzyme-system-three-cellulase gene (*sestc*) was genetically integrated into the *S. cerevisiae* genome using the protoplast method. Several recombinant strains of *S. cerevisiae*, capable of expressing cellulase with high efficiency, were selected. The total activity of cellulase, endo-β-D-glucanase, exo-β-D-glucanase, and xylanase of the recombinant *S. cerevisiae* transformant number 14 were 1.1, 378, 1.44, and 164 U ml^−1^, respectively, which were 27.5-, 63-, 24-, and 19-fold higher than those of the control. The concentrations of ethanol produced by the engineered *S. cerevisiae* strain and the wild-type were 8.1 and 0.14 gl^−1^, with wheat bran as the carbon source, under submerged conditions.

This study provided an alternative method for the production of bio-ethanol by adopting the mechanism of consolidated bioprocessing without the addition of the cellulase and xylanase derived from other microorganisms. This method achieved true in situ saccharification and fermentation and would thus substantially save costs during the cellulase production and saccharification process. However, some potential limitations should first be addressed, such as the difference in optimal temperature between cellulase production by the fermentation and saccharification process and low expression activity of the cellulase gene leading to low conversion efficiency of lignocellulosic bio-ethanol. Further studies should focus on the domestication of an engineered strain to increase cellulase activity, optimization of the promoter to promote the expression of heterogeneous genes, and thermal tolerance of *S. cerevisiae* at 50 °C to enhance the application value of engineered strains of *S. cerevisiae*.
